# Interdental papilla recession and reconstruction of the lost triangle: a review of the current literature

**DOI:** 10.3389/fdmed.2024.1537452

**Published:** 2025-01-17

**Authors:** Sanabel O. Barakat

**Affiliations:** Department of Oral & Maxillofacial Surgery, Faculty of Dentistry, Zarqa University, Zarqa, Jordan

**Keywords:** interdental papilla, IDP recession, interdental papilla reconstruction, GBT, IDP, receded papilla, gingival black triangle

## Abstract

Interdental papilla (IDP) deficiency and the presence of gingival black triangles (GBT) are major concerns for both patients and dentists, as the IDP plays an important role in esthetics due to its strong association with the patient's smile. Interdental papilla deficiency is frequent among different populations, with a tendency to increase with age and in patients with periodontal disease. In addition, GBT causes phonetic problems, food impaction, plaque accumulation, and increased risk for root caries. The small dimensions of the IDP and the limited vascular supply to the interproximal space render treatment modalities of receded papillae unpredictable. Still, and based on the etiological factors, several non-surgical treatment options, including correction of traumatic oral hygiene practices, restorative interventions, papilla priming, papilla enhancement with either autologous fibroblast injection or hyaluronic acid, and orthodontic therapy, have been proposed to fill the GBT. In addition, different surgical techniques—with or without grafting biomaterials—have also been introduced to reconstruct the lost papilla. Nonetheless, there is no gold standard set yet. Further, systematic reviews evaluating the efficacy of surgical reconstruction of deficient IDP are still lacking due to the scarcity of large-scale clinical trials and the absence of long-term clinical outcomes. The aim of this review was to identify various causes of IDP recession as well as to explore the available treatment modalities to reconstruct the lost papilla.

## 1 Introduction

The interdental papilla (IDP) is the extension of the gingiva filling the spaces between adjacent teeth. It extends from the tip of the papilla to a line tangential to the gingival margin of the two adjacent teeth (1). In health, the embrasure will house the entire papilla, leaving no space between the tip of the papilla and the contact point (2).

An intact gingival unit is one of the key factors in maintaining esthetics. The interdental soft tissues play a critical role in what constitutes pink esthetics and an esthetic smile (3, 4).

The absence of the IDP from its embrasure or the presence of space apical to the interproximal contact point is defined as IDP deficiency or recession (5). IDP is not uncommon among adult populations. The prevalence of gingival black triangles (GBT) increases significantly with age and in the presence of periodontal disease (6). This condition is not only perceived by dental professionals as unesthetic, but also by the lay public. Kokich et al. (7) reported that both dentists and patients described GBT greater than 3 mm as “less attractive.” In addition, Cunliffe and Prety (8) reported that patients ranked GBT as the third most disliked esthetic problem after caries and visible crown margins.

## 2 Factors affecting the presence of IDP

### 2.1 Bone crest to interproximal contact point distance

In a landmark study, Tarnow et al. (9) described the “5 mm” rule that governs the presence of IDP. They studied the presence of IDP in relation to the vertical distance between the interproximal bone crest and the contact point, using periodontal sounding. The authors reported that papilla was present in 98% of sites when the vertical distance between the contact point and crest of bone was ≤5 mm. When the distance was 6 mm, IDP was present in 56% of the cases. However, when the distance increased to 7 mm, the papilla was mostly missing (73%). Consistently, Wu et al. (10) reproduced Tarnow's findings. Cho et al. (11) reported that the presence of interdental papillae decreased as the bone-to-contact distance increased from approximately 90% of sites measuring ≤4 mm, to 58.5% in ≤5 mm sites, and 35% in 6 mm sites. Further, Chen et al. (12) indicated that IDPs are more likely to exist in the shorter distance between the alveolar bone crest and the contact point and in smaller embrasures. IDP was present in 100% of cases when the distance was ≤4 mm and in approximately 80% of cases when the distance was ≤5 mm.

### 2.2 Gingival biotype and crown morphology

Ahmad (13) considered the flat biotype as more favorable to achieving papilla fullness compared to thin scalloped tissue, where adjacent triangular teeth have a more coronally positioned contact point, resulting in an increased distance between the contact point and alveolar crest, which, in turn, predisposes to IDP recession. On the other hand, Rafiee and Melamed (14) did not find any significant association between gingival biotype and the presence of complete IDP in their results.

In this regard, Ziahosseini et al. (15) indicated that thin scalloped tissue is more likely to react to trauma or inflammation by recession while thick flat tissue reacts with deeper periodontal pockets. They added that the restricted blood supply in the thin biotype, at the papilla tip, can reduce vascularity and interrupt the healing of an injured IDP, resulting in unpredictable repair and thus recession, whereas thicker tissues respond more favorably due to their increased vascularity because they can cope better with inflammatory responses. Moreover, Jamwal et al. (16) reported that a square crown yields better interdental papilla maintenance due to wider contact and a smaller interproximal distance from the osseous crest to the contact point, while the triangular crown form is associated with high pronounced gingival scallop and thin underlying crestal bone, which predisposes for IDP recession.

### 2.3 Periodontal disease and trauma

The interdental area was described as the most liable part of the periodontium to plaque accumulation gingivitis and periodontal disease. Its anatomy combined with the vascular supply allows for periodontal disease to progress rapidly in this area (17). Interproximal bone resorption secondary to periodontal disease causes the development of black triangles (18).

Mechanical trauma to the IDP might result in open embrasures (19). Moreover, faulty oral hygiene measures or improper use of interdental aids, such as traumatic flossing or aggressive bushing, may contribute to open embrasures (20).

### 2.4 Interproximal distance between adjacent teeth

Cho et al. (11) reported that the number of papillae that filled the interproximal space decreased as the distance between adjacent roots increased; when the interradicular distance was 1 mm, 77.8% of IDP were fully present. This proportion decreased gradually until papillae were totally lost where the distance exceeded 3.5 mm. A very wide interdental width increases the risk of the presence of a GBT, as the presence of diastemas may present lack of teeth support resulting in reduced papilla height.

### 2.5 Patients’ age

Although van der Velder (21) did not find sufficient evidence for physiological gingival or papillae apical migration during aging, Chow et al. (22) reported that papilla height decreased with time and the probability of finding open embrasures increased with age, at a rate of 0.012 mm/year. Chang (5) also found that papilla recession was associated with older age. Billings et al. (23) also reported that interproximal sites were increasingly affected by recession as age increased while probing depths remained relatively stable. It was explained that aging results in the thinning of oral epithelium, decreased keratinization, reduced papilla height, and bone loss, which are all risk factors for GBT (15).

## 3 Treatment of IDP recession

### 3.1 Non-surgical treatment

#### 3.1.1 Periodontal approach

When the interdental papilla is associated with periodontal disease, its recovery may be expected after non-surgical periodontal treatment. Yanagishita et al. (24) reported an improvement of receded interdental papillae in a periodontal patient who presented with widespread gingival and papilla recessions. After initial periodontal treatment and oral hygiene instructions, a gradual improvement in the papillae height was observed together with coronal regrowth of the gingival margin in this patient.

Papilla recession, induced by traumatic oral hygiene practices, may be treated by initially stopping interproximal oral hygiene practices to allow time for the injured papilla to heal and later on by modifying oral hygiene measures practiced by the patient (25).

#### 3.1.2 Orthodontic and restorative approaches

It is believed that orthodontic treatment is effective in promoting the vertical growth of the existing interdental soft tissue to eliminate GBT. Cardaropoli et al. (26) demonstrated that continuous pressure on teeth surrounded by open embrasures resulted in the closure of interdental space. In addition, Al-Zarea et al. (27) reported that conventional orthodontic movement brings separated adjacent teeth closer to squeeze the papillary soft tissue to move it coronally and a new contact point or area may be created. However, the teeth should not be moved too close together because close proximity of the roots could increase the risk of bone resorption and jeopardize the integrity of the papilla (28).

Restorative techniques and prosthetic work do not reconstruct papillary defects but merely mask GBT lengthening interproximal contacts (16, 29).

#### 3.1.3 Repeated curettage

In a case report, Shapiro (30) described performing repeated scaling/root planning and curettage of the papillary tissue every 15 days for 3 months. After 9 months of initial treatment, a reduction in GBTs was observed. It was suggested that instrumentation may have induced a proliferative hyperplastic inflammatory reaction of the papilla since some newly regenerated papillae were observed (17, 31).

#### 3.1.4 Tissue volumizing

##### 3.1.4.1 Hyaluronic acid

Hyaluronic acid (HA) is a major tissue filler that been introduced for the management of a receded IDP (32). Becker et al. (33) suggested enhancement of interdental papillae that do not entirely fill the interdental space with HA. They injected HA in receded papillae in the esthetic zone three times at intervals of 3 weeks. According to the results, half of the sites showed a 57%–97% fill of the GBTs. In another study, Singh and Vandana (34) tested three concentrations of HA gel in the enhancement of deficient IDPs. They used 1%, 2%, and 5% HA. The results indicated that 5% of HA gel showed highly significant papilla enhancement (*p* = 0.001) at 6 months compared with lower concentrations.

In this regard, Sanchez-Perez et al. (35) concluded that the HA injections were effective for the reconstruction of IDP defects within a period of 6 months, if the distance between the contact point and the bone crest is <6 mm. Nonetheless, the average height increase achieved was only 0.43 mm.

##### 3.1.4.2 Autogenous fibroblasts

McGuire and Scheyer (36) harvested autogenous fibroblasts from the maxillary tuberosity areas from patients with deficient IDPs. The cells were expanded and prepared for injection. One week after the initial priming procedure, the receded papillae received an injection of autologous fibroblasts. Two more injections were performed after 7–14 days. According to the results, the treatment areas showed a statistically significant mean increase in papilla height of approximately 0.3 mm (*p* = 0.0006) after 4 months.

##### 3.1.4.3 Stem cells

Yamada et al. (37) used autogenous bone marrow mesenchymal cells harvested from the patient’s iliac crest 1.5 months before IDP reconstruction and the aspirate was cultured in serum to be extended. Platelet-rich plasma from the patient's blood was used as a source for growth factors and HA was the scaffold in this procedure. For interproximal tissue augmentation, the mixture was injected adjacent to the reduced IDP and the patients were followed up for 55 months. According to the results, a mean increase in IDP height of 2.55 ± 0.89 mm may be achieved in a tissue engineered papilla.

### 3.2 Surgical treatment

#### 3.2.1 Papilla preservation technique

Takei et al. (38) introduced the conventional papilla preservation technique (PPT). In this flap, sulcular incisions around each tooth with palatal flap involving a semilunar incision were made across the IDP. The incision dipped apically from the line angles of the tooth so that the papillary incision line angle was at least 5 mm from the gingival margin, exposing the bone defect and interdental tissues to be dissected from the lingual or palatal aspect so that it could be elevated intact with a facial flap. At least 2 mm of papillary tissue was maintained. Then, intrabony defect debridement was made to remove all granulation tissue. Later, the bone graft was placed and the flap was sutured. According to the report, wound healing always occurred by primary intention and without evidence of immediate graft exfoliation. Interdental soft tissue craters did not develop, which made it easier for the patients to practice optimal oral hygiene.

According to Jenabian et al. (39), this procedure was found to be comparable with other papilla preservation surgical techniques, especially when a connective tissue graft (CTG) was used to augment the lost tissue. Although it was originally prescribed to address interproximal intrabony defects, it may be suitable to treat IDP soft tissue deficiency (20).

#### 3.2.2 Beagle technique

Beagle (40) presented a case report to describe a pedicle graft procedure utilizing the soft tissues palatal to the interdental area. In this technique, a split-thickness flap was dissected on the palatal aspect of the interdental area. Then, a partial thickness incision was made along the line angles of adjacent teeth on the palatal aspect. Sulcular incisions were given in the interdental region to separate the papillary unit. The partial thickness flap was then elevated to the labial aspect and the elongated papilla was folded on itself, to approximate the connective tissue sides, to create the new papilla at the facial part of the interdental area. A periodontal dressing was applied on the palatal aspect only, to support the papilla.

#### 3.2.3 Modified Beagle’s technique

In this technique, unlike Beagle's, incisions were given buccally at the adjacent line angles of the interdental papilla, such that the length was longer than the length of the black triangle space to be reconstructed. These vertical incisions were connected by a horizontal incision at the apical end. A partial thickness pedicle flap was then elevated. The papillary unit was carefully dissected from the teeth and the interdental area so that the papillary unit could be mobilized freely. The pedicle graft was then advanced coronally to completely obliterate the black triangle space. The flap was then sutured coronally to the adjacent tissues and no periodontal dressing was used (41).

Comparing the Beagle and modified Beagle surgical techniques, Chaulkar et al. (42) conducted a randomized clinical trial that included 20 sites with papilla recession in the maxillary anterior region. According to the results, at 6 months postoperatively, there was a statistically significant bigger reduction in the vertical and mesiodistal dimensions, as well as the area of the GBT in the modified Beagle group compared to the Beagle technique group.

#### 3.2.4 Han and Takei technique

Han and Takei (43) proposed the “semilunar coronally repositioned papilla” to reconstruct papilla loss due to soft tissue damage. In their description, a semilunar incision was placed in the alveolar mucosa facial to the interdental area and a pouch-like preparation was performed in the interdental area. Intrasulcular incisions were made around the mesial and distal halves of the two adjacent teeth to free the connective tissue from the root surfaces to allow coronal displacement of the gingival–papillary unit. A CTG, harvested from the palate, was placed into the pouch to support the interdental tissue. The integrity of the IDP was maintained by moving the whole gingival–papillary unit coronally.

In this regard, Shruthi et al. (44) compared Han and Takei's semilunar incision with Azzi's technique in reconstructing the lost central IDP. Their results showed a statistically significant improvement in papilla presence index score and in papillary height from baseline, with no significant difference between the study groups.

The Han and Takei technique was also compared to the PPT for IDP reconstruction, using a CTG. Both techniques had a positive effect on papilla height with no significant difference in outcomes found between the techniques (39).

Han and Takei's surgical technique may be effective in restoring papilla height even when used with platelet concentrates. In a recent randomized clinical trial, Barakat et al. (45) used this procedure to reconstruct receded papillae. For this purpose, the researchers compared the effect of advanced platelet-rich fibrin (A-PRF) to CTG as augmenting biomaterials. After a follow-up period of 12 months, a mean gain in papilla height of 2.25 ± 0.97  mm in the CTG group and of 1.86 ± 0.7 mm in the A-PRF group was reported, with no significant difference (*p* = 0.206) between the study groups. From this experiment, it was concluded that both CTG and A-PRF are equally effective in increasing deficient IDP height when used with Han and Takei's approach.

#### 3.2.5 Azzi technique

An envelope-type flap procedure was proposed by Azzi et al. (46) to surgically restore the lost IDP. This envelope was prepared to cover a connective tissue graft to augment the deficient papilla. An intrasulcular incision was made at the tooth surfaces facing the interdental area to be reconstructed. Subsequently, an incision was made across the facial aspect of the interdental area and an envelope-type flap was elevated into the proximal site as well as apically to a level beyond the mucogingival line. A CTG was harvested and placed under the flaps in the interdental papilla area. The flaps were then brought together and sutured. Azzi's approach showed satisfactory results when used in the treatment of IDP recession (44, 47, 48).

#### 3.2.6 The tube technique

In their case report, Kashani et al. (49) described the tube technique for papilla reconstruction. In this procedure, a semilunar incision was placed at the buccal aspect at the level of the mucogingival junction and a full thickness flap was elevated. The flap was extended mesiodistally by an additional 4–5 mm than needed for the graft. Another semilunar incision was placed on the palatal aspect in a way so that the zenith of the incision was located proximal to the alveolar crest. The papillary tissue was then reflected away from the root surface and alveolar bone. The recipient site at this stage resembled a tube with two openings. The harvested connective tissue graft was inserted into this tube to augment the deficient tissue interproximally. This report indicated a net gain in the interproximal tissues of 4–5 mm.

## 4 Discussion

Patients may present one or more etiological factors for open embrasures; therefore, managing each patient requires individual assessment and a customized treatment plan (50). In many cases, a multidisciplinary approach may be considered (51, 52).

Various surgical flap designs have been used to access the embrasures. While some clinicians favored tunneling to raise the deficient papilla, others implemented flap mobilization after papilla incision designs (53). Selecting the most appropriate surgical technique for restoring a lost IDP requires consideration of several factors, including flap design, surgical approach, periodontal status, and the presence of gingival recession.

It is noteworthy that despite the availability of several surgical treatment options for IDP augmentation, there is no gold standard set yet (54) due to the lack of large-scale clinical trials and the absence of long-term clinical outcomes (18, 51). Nonetheless, CTG-based surgical approaches have shown promising results (39), similar to other mucogingival surgeries and gingival augmentation procedures (55).
